# Supplementary material for: Role of hematological parameters in the early detection of clinical cases for septicemia among neonates: A hospital-based study from Chennai, India
Source: PLoS One. 2025 Mar 19;20(3):e0318802. doi: 10.1371/journal.pone.0318802 (PMC11922526; doi:10.1371/journal.pone.0318802)
Supplement: Attachment — Submitted filename: PONE-D-24-54291.pdf [file pone.0318802.s001.pdf]

Manuscript Number: PONE-D-24-54291

Title: Role of hematological parameters in the early detection of clinical cases for septicemia among neonates: A hospital-based study from Chennai, India.

Overview and general recommendation:

This subject is important because there is a significant need for accurate and quick diagnostic methods to identify newborns at risk of sepsis and commence prompt therapies.

I found the manuscript well written, well described. I listed **minor points** below for editing.

Comments to Authors:

Minor points

1- In Materials and Methods (136 line) and in results (202line); you wrote (2 times We)! The rule of manuscript writing is to avoid using (We). So you should delete (We) and use academic scientific words such as (This study or The current study or The present study).

2- In results, 211line; you wrote **TLC!!!** you should write the whole name of the term; **total leukocyte count (TLC)** not only the abbreviation to be obvious to the reader because here you used this term for the first time while in next sentences you can use the abbreviation only.

3- In discussion; 293line, 295line, 300line, 303line, 310line, 325line, 329line, 333line, 341line, 342line and 380 line in conclusion; you wrote (11 times Our)! The rule of manuscript writing is to avoid using (Our). So you should delete (**Our**) and use academic scientific words **such as** (**This study or The current study or The present study**).

4- In references, the old references are more than the new references!!! Why!!! Kindly solve this point.

Kind regards
