# Supplementary material for: Role of hematological parameters in the early detection of clinical cases for septicemia among neonates: A hospital-based study from Chennai, India
Source: PLoS One. 2025 Mar 19;20(3):e0318802. doi: 10.1371/journal.pone.0318802 (PMC11922526; doi:10.1371/journal.pone.0318802)
Supplement: Attachment — Submitted filename: Rebuttal letter- Plos One.docx [file pone.0318802.s003.docx]

Dear Editor,

We wish to convey our profound gratitude for the scrupulous assessment of our manuscript and for offering intellectual recommendations aimed at its refinement. Each suggestion has been diligently reviewed, and the requisite amendments have been meticulously integrated into the revised manuscript, as demonstrated through the tracked changes.

Enclosed below are our comprehensive responses, accompanied by justifications, addressing each comment provided by the editor and reviewers:

**Editorial Comments:**

***Comment 1:*** *Please ensure that your manuscript meets PLOS ONE's style requirements, including those for file naming. The PLOS ONE style templates can be found at*

[*https://journals.plos.org/plosone/s/file?id=wjVg/PLOSOne_formatting_sample_main_body.pdf*](https://journals.plos.org/plosone/s/file?id=wjVg/PLOSOne_formatting_sample_main_body.pdf)

[*https://journals.plos.org/plosone/s/file?id=ba62/PLOSOne_formatting_sample_title_authors_affiliations.pdf*](https://track.editorialmanager.com/CL0/https:%2F%2Fjournals.plos.org%2Fplosone%2Fs%2Ffile%3Fid=ba62%2FPLOSOne_formatting_sample_title_authors_affiliations.pdf/1/010f0193f5ca4011-a935d75c-d01d-4e19-bc0a-9b073df171cc-000000/1QXW36FTWeZm6xPZrY0I4mCUZLS_y9KeaooCtoEXce0=190)

**#Author’s response:** The manuscript has been meticulously reformatted to adhere to the prescribed style and formatting requirements of PLOS ONE.

***Comment 2:*** *In the ethics statement in the Methods, you have specified that verbal consent was obtained. Please provide additional details regarding how this consent was documented and witnessed, and state whether this was approved by the IRB.*

**#Author’s response:** As per your recommendation, the ethics statement in the Methods section has been elaborated upon to detail the procedures for obtaining and documenting verbal consent, along with the role of witnesses. Furthermore, approval from the Institutional Review Board (IRB) has been explicitly stated.

***Comment 3.*** *We note that your Data Availability Statement is currently as follows: “All relevant data are within the manuscript and in Supporting Information files”*

*Please confirm at this time whether or not your submission contains all raw data required to replicate the results of your study. Authors must share the “minimal data set” for their submission. PLOS defines the minimal data set to consist of the data required to replicate all study findings reported in the article, as well as related metadata and methods (*[*https://journals.plos.org/plosone/s/data-availability#loc-minimal-data-set-definition*](https://journals.plos.org/plosone/s/data-availability#loc-minimal-data-set-definition)*).*

*For example, authors should submit the following data:*

*- The values behind the means, standard deviations and other measures reported;*

*- The values used to build graphs;*

*- The points extracted from images for analysis.*

*Authors do not need to submit their entire data set if only a portion of the data was used in the reported study.*

*If your submission does not contain these data, please either upload them as Supporting Information files or deposit them to a stable, public repository and provide us with the relevant URLs, DOIs, or accession numbers. For a list of recommended repositories, please see* [*https://journals.plos.org/plosone/s/recommended-repositories*](https://journals.plos.org/plosone/s/recommended-repositories)*.*

*If there are ethical or legal restrictions on sharing a de-identified data set, please explain them in detail (e.g., data contain potentially sensitive information, data are owned by a third-party organization, etc.) and who has imposed them (e.g., an ethics committee). Please also provide contact information for a data access committee, ethics committee, or other institutional body to which data requests may be sent. If data are owned by a third party, please indicate how others may request data access.*

**#Author’s response:** The Data Availability Statement has been amended to read as: ‘Public sharing of the data is restricted in accordance with the policies of the Neonatal Intensive Care Unit (NICU) at Saveetha Medical College and Hospital. Permission for data usage has been granted by the hospital administration strictly for research purposes, adhering to institutional regulations and ethical standards.’

***Comment 4:*** *When completing the data availability statement of the submission form, you indicated that you will make your data available on acceptance. We strongly recommend all authors decide on a data sharing plan before acceptance, as the process can be lengthy and hold up publication timelines. Please note that, though access restrictions are acceptable now, your entire data will need to be made freely accessible if your manuscript is accepted for publication. This policy applies to all data except where public deposition would breach compliance with the protocol approved by your research ethics board. If you are unable to adhere to our open data policy, please kindly revise your statement to explain your reasoning and we will seek the editor's input on an exemption. Please be assured that, once you have provided your new statement, the assessment of your exemption will not hold up the peer review process.*

**#Author’s response:** We have not selected any option for this question, as sharing data is currently restricted by institutional policies.

***Comment 5:*** *Please include captions for your Supporting Information files at the end of your manuscript, and update any in-text citations to match accordingly. Please see our Supporting Information guidelines for more information:* [*http://journals.plos.org/plosone/s/supporting-information*](http://journals.plos.org/plosone/s/supporting-information)*.*

**#Author’s response:** Since our manuscript does not include Supporting Information files, this requirement is not applicable.

**Additional Editor’s Comments:**

***Comment 1:*** *Line 79-80 “Early-onset newborn infections of viral or fungal origin may also arise at seven days of life and must be differentiated from bacterial sepsis”: more references are needed. Please cite more references, with the following one as an example (citing is optional):*

*Liu BM, Mulkey SB, Campos JM, DeBiasi RL. Laboratory diagnosis of CNS infections in children due to emerging and re-emerging neurotropic viruses. Pediatr Res. 2024 Jan;95(2):543-550. doi: 10.1038/s41390-023-02930-6. Epub 2023 Dec 2. PMID: 38042947.*

**#Author’s response:** Suggested reference and its associated text content has been added in the revised manuscript.

***Comment 2:*** *Line 87-88 “Blood culture still remains the gold standard for diagnosing sepsis, but it is tedious (at least 24-48 hours) and necessitates a well-equipped laboratory”: more references are needed. Please cite more references, with the following one as an example (citing is optional):*

*Liu BM, Carlisle CP, Fisher MA, Shakir SM. The Brief Case: Capnocytophaga sputigena Bacteremia in a 94-Year-Old Male with Type 2 Diabetes Mellitus, Pancytopenia, and Bronchopneumonia. J Clin Microbiol. 2021 Jun 18;59(7):e0247220. doi: 10.1128/JCM.02472-20. Epub 2021 Jun 18. PMID: 34142857; PMCID: PMC8218739.*

**#Author’s response:** Suggested reference and its associated text content has been added in the revised manuscript.

***Comment 3:*** *Line 88-89 “the diagnosis should be done as early as possible to start the treatment”: more references are needed. Please cite more references, with the following one as an example (citing is optional):*

*Liu B, Forman M, Valsamakis A. Optimization and evaluation of a novel real-time RT-PCR test for detection of parechovirus in cerebrospinal fluid. J Virol Methods. 2019 Oct;272:113690. doi: 10.1016/j.jviromet.2019.113690. Epub 2019 Jul 5. PMID: 31283959.*

**#Author’s response:** Suggested reference and its associated text content has been added in the revised manuscript.

***Comment 4:*** *Line 372-374: “Advances in rapid diagnostic technologies, particularly molecular assays and point-of-care testing, show promise for enhancing diagnosis accuracy and accelerating treatment interventions in newborns”: more references are needed. Please cite more references, with the following one as an example (citing is optional):*

*Liu BM. Epidemiological and clinical overview of the 2024 Oropouche virus disease outbreaks, an emerging/re-emerging neurotropic arboviral disease and global public health threat. J Med Virol. 2024 Sep;96(9):e29897. doi: 10.1002/jmv.29897. PMID: 39221481.*

**#Author’s response:** Suggested reference and its associated text content has been added in the revised manuscript.

***Comment 5:*** *Line 375- 376 “specialized research concentrating on host-response biomarkers and microbial genetics may further improve diagnostic algorithms and increase individualized treatment options”: more references are needed. Please cite more references, with the following ones as example (citing is optional):*

*Liu BM, Li NL, Wang R, Li X, Li ZA, Marion TN, Li K. Key roles for phosphorylation and the Coiled-coil domain in TRIM56-mediated positive regulation of TLR3-TRIF-dependent innate immunity. J Biol Chem. 2024 May;300(5):107249. doi: 10.1016/j.jbc.2024.107249. Epub 2024 Mar 29. PMID: 38556084; PMCID: PMC11067339.*

*Liu BM, Mulkey SB, Campos JM, DeBiasi RL. Laboratory diagnosis of CNS infections in children due to emerging and re-emerging neurotropic viruses. Pediatr Res. 2024 Jan;95(2):543-550. doi: 10.1038/s41390-023-02930-6. Epub 2023 Dec 2. PMID: 38042947.*

**#Author’s response:** Suggested reference and its associated text content has been added in the revised manuscript.

**Reviewer’s Comments to the Author:**

**Reviewer #1:**

*Minor points:*

***Comment 1:*** *In Materials and Methods (136 line) and in results (202line); you wrote (2
times We)! The rule of manuscript writing is to avoid using (We). So you
should delete (We) and use academic scientific words such as (This study or
The current study or The present study).*

**#Author’s response:** We greatly appreciate this observation. The suggested revisions have been implemented, replacing "We" with formal expressions such as "This study" or "The present study."

***Comment*** *2: In results, 211 line; you wrote TLC!!! you should write the whole name of
the term; total leukocyte count (TLC) not only the abbreviation to be
obvious to the reader because here you used this term for the first time while
in next sentences you can use the abbreviation only.*

**#Author’s response:** Thank you for highlighting this point. The correction has been made by explicitly defining "total leukocyte count (TLC)" at its first mention.

***Comment 3:*** *In discussion; 293 line, 295line, 300line, 303line, 310line, 325line, 329line,
333line, 341line, 342line and 380 line in conclusion; you wrote (11 times
Our)! The rule of manuscript writing is to avoid using (Our). So you should
delete (Our) and use academic scientific words such as (This study or The
current study or The present study).*

**#Author’s response:** We acknowledge this valuable suggestion. All instances of "Our" have been replaced with more formal alternatives, such as "This study" or "The present study."

***Comment 4:*** *In references, the old references are more than the new references!!! Why!!!
Kindly solve this point.*

**#Author’s response:** As suggested, older references have been replaced or supplemented with more recent citations, ensuring a balanced representation of current literature.

**Reviewer #2:**

***Comment 1:*** *Regarding background of abstract must be changed as the title is hematological changes in neonatal sepsis !!! why in the background highlighting blood culture , better to replaced by hematological finding or even start with pathogenesis of sepsis.*

**#Author’s response:** We greatly appreciate this insightful recommendation. The background section of the abstract has been revised accordingly in the updated manuscript.

***Comment 2:*** *Both table 6 & 7 should be rearranged based on gram positive isolates and gram negative isolates not mixing together.*

**#Author’s response:** We greatly appreciate this insightful recommendation. The background section of the abstract has been revised accordingly in the updated manuscript.
